# Supplementary material for: Van der Waals β-Ga2O3 thin films on polycrystalline diamond substrates
Source: Nat Commun. 2025 Aug 31;16:8144. doi: 10.1038/s41467-025-63666-x (PMC12398616; doi:10.1038/s41467-025-63666-x)
Supplement: Supplementary file 1 — Supplementary Information [file 41467_2025_63666_MOESM1_ESM.pdf]

Van der Waals β-Ga_2O_3 Thin Films on Polycrystalline Diamond Substrates

Jing Ning[1, 2, 3, #, *], Zhichun Yang[1, 2, #], Haidi Wu[1, 2], Xinmeng Dong[1, 2], Yaning Zhang[1, 2], Yufei Chen[1, 2], Xinbo Zhang[1, 2], Dong Wang[1, 2, 3], Yue Hao[1, 2, 3], Jincheng Zhang[1, 2, 3,*]

[1]The State Key Laboratory of Wide-Bandgap Semiconductor Devices and Integrated Technology, Xi'an 710071, China;

[2]Shaanxi Joint Key Laboratory of Graphene, Xidian University, Xi'an 710071, China;

[3]Xidian-Wuhu Research Institute, Xidian University, Wuhu 241000, China.

# J. Ning and Z. Yang contributed equally to this manuscript.

*Corresponding author. Email: ningj@xidian.edu.cn (J. Ning)

jchzhang@xidian.edu.cn (J. C. Zhang)

1. Cross-sectional TEM micrographs revealing interfacial structures.

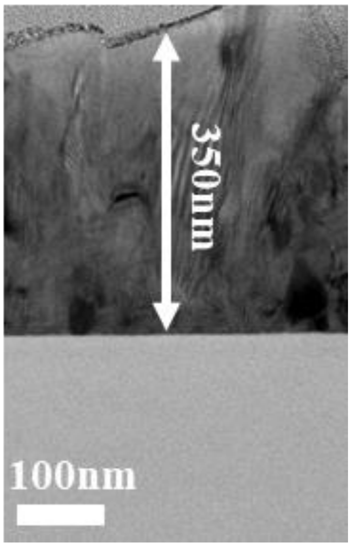

Supplementary Fig. 1 High-magnification cross-sectional TEM image of the interface between β-Ga_2O_3, graphene, and poly-diamond

2. XPS Temporal Stability Validation

We performed new comparative XPS measurements between freshly prepared samples and those stored under ambient conditions for 48 hours. Spectral deconvolution of the O1s peaks reveals that Olat corresponds to lattice oxygen in Ga_2O_3 while O_{Vac} relates to oxygen vacancies. Quantitative analysis shows the oxygen vacancy concentration $O_{Vac}/(O_{Lat} + O_{Vac})$ decreased marginally from 32.8% (fresh) to 30.6% (48-hour ambient storage), confirming only superficial oxidation that doesn't alter the fundamental oxygen vacancy trends reported in our original findings.

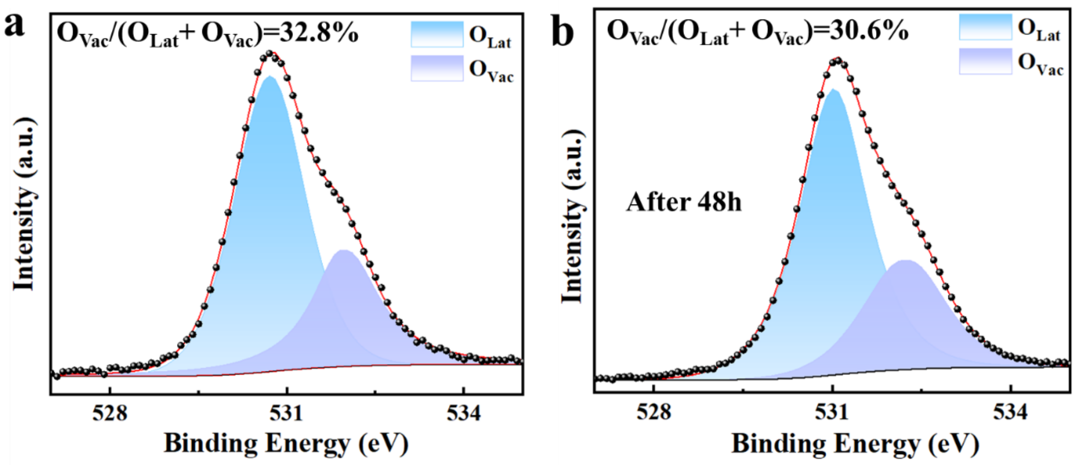

Supplementary Fig. 2 O1s XPS spectra of **a,** as-grown β-Ga_2O_3 and **b,** after 48-hour ambient air exposure. Source data are provided as a Source Data file.

3. The wet transfer method of Graphene onto polycrystalline diamond

Prior to graphene transfer onto the polycrystalline diamond substrate, the diamond surface underwent sequential ultrasonic cleaning with acetone, ethanol, and deionized water, followed by immersion in dilute HF solution for 5 minutes to remove surface oxides, ensuring interfacial cleanliness. The AFM and SEM images of graphene on the polycrystalline diamond surface are shown in **Supplementary Fig. 3a** and **b**. The polycrystalline diamond substrate exhibits random orientations with clearly visible grain boundaries, demonstrating an RMS roughness of approximately 0.3 nm. Due to the influence of this polycrystalline substrate, the wet-transferred graphene shows varying degrees of wrinkles with an RMS roughness of about 0.51 nm. The SEM images confirm the graphene film is free from macroscopically visible cracks, supporting its low-defect characteristics.

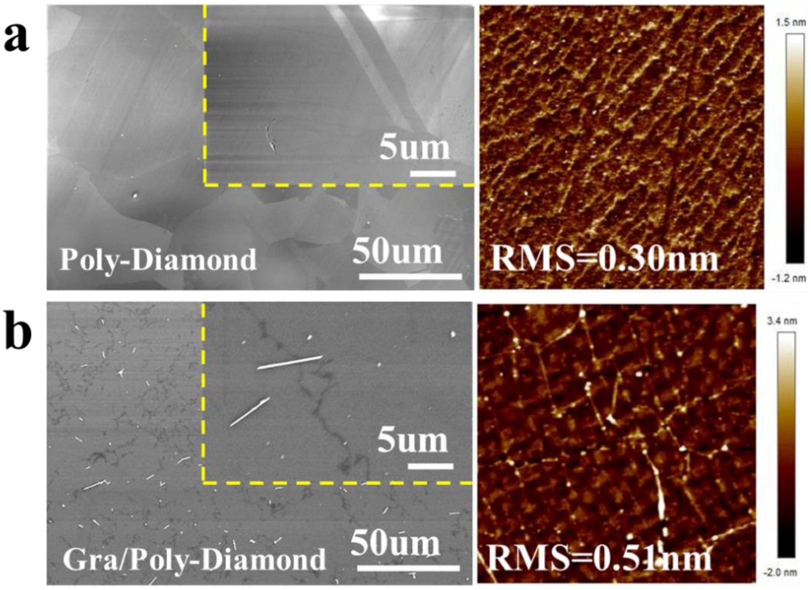

Supplementary Fig. 3 a, SEM and AFM images of the polycrystalline diamond substrate. **b,** SEM and AFM images of wet-transferred monolayer graphene on polycrystalline diamond.

4. Cross-sectional SEM images revealing grain boundary formation conditions in the thin films.

As shown in **Supplementary Fig. 4a**, SEM images before and after Ga_2O_3 growth on graphene/poly-diamond substrates reveal that wrinkles in the underlying graphene layer led to variations in the Ga_2O_3 growth rate, resulting in regions with varying contrast. Similarly, gas flow can have a comparable effect. As illustrated in **Supplementary Fig. 4b**, under higher gas flow rates, reactive gases or precursors may be rapidly carried away before sufficient adsorption on the substrate surface occurs, leading to unstable deposition rates and even non-uniform deposition regions. Conversely, if the gas flow rate is too low, it may result in insufficient supply of reactive gases or overly slow deposition rates. In our experiment, an RMS roughness as high as 51.3 nm was observed at a flow rate of 300 sccm. Therefore, in practical applications, it is essential to optimize an appropriate range of gas flow rates to ensure high-quality thin film deposition.

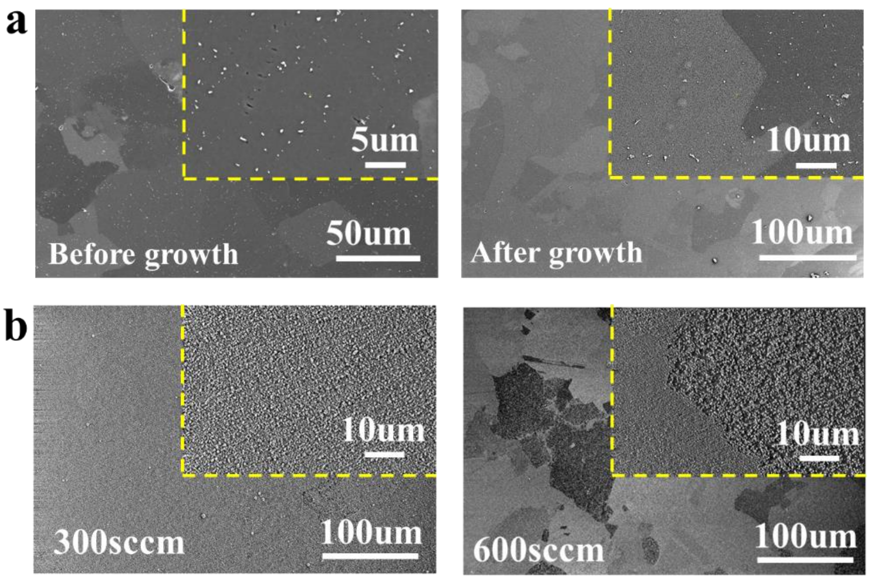

Supplementary Fig. 4 a, SEM images of β-Ga_2O_3 before and after van der Waals epitaxy on graphene/poly-diamond. **b,** SEM images of epitaxial β-Ga_2O_3 films grown under O_2 flow rates of 300 and 600 sccm.

5. Temperature-dependent Raman peak shift analysis

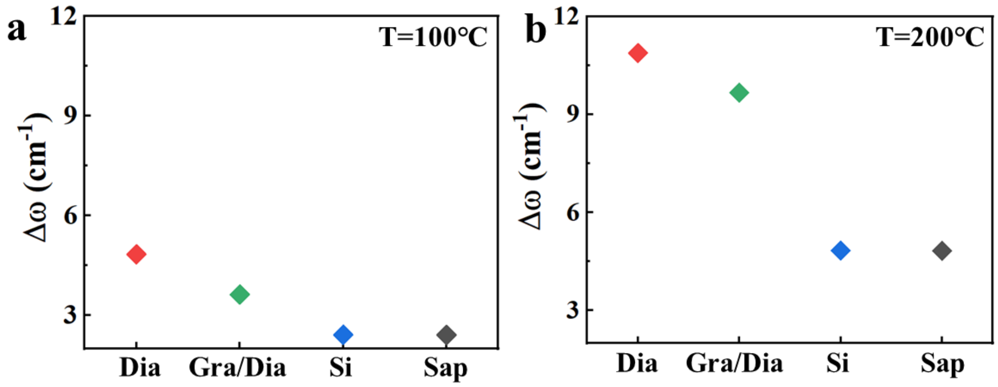

Supplementary Fig. 5 Comparative data of Raman peak red shifts (Δω) in β-Ga_2O_3 on different substrates at **a,** 100°C and **b,** 200°C, respectively. Source data are provided as a Source Data file.

6. The long-term stability of photodetectors

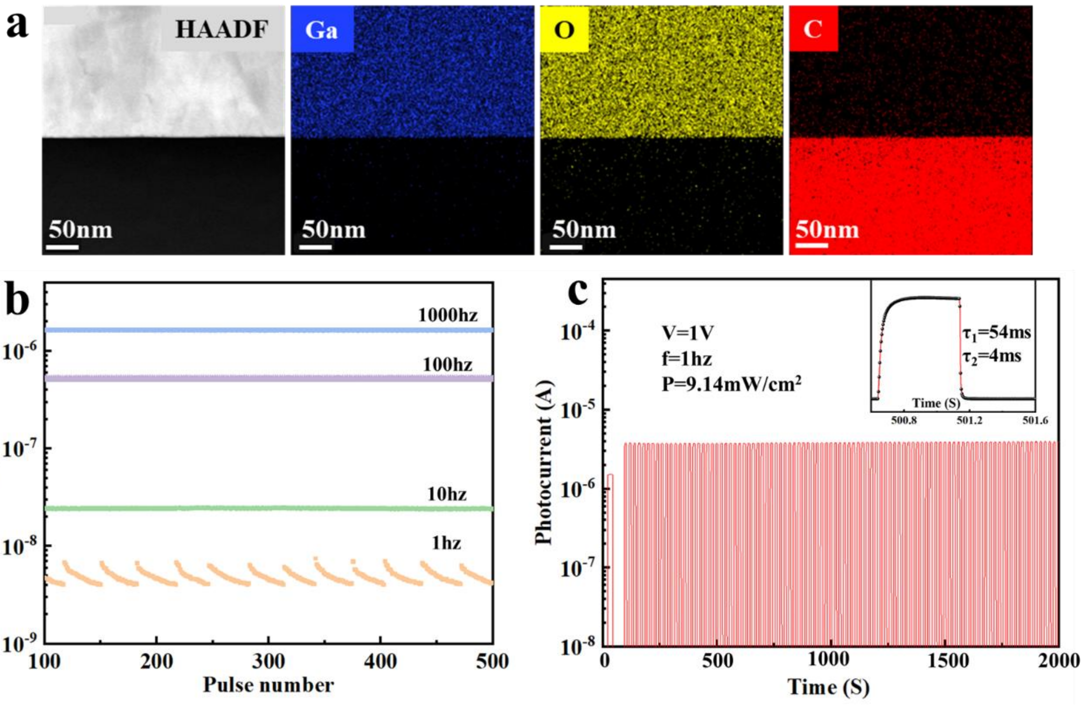

Supplementary Fig. 6 a, Cross-sectional EDS elemental mapping of the heterointerface. **b,** Frequency-dependent conductivity response (1 Hz-1000Hz) under pulsed optical excitation. **c,** Long-term photocurrent stability test spanning 2000 s under continuous 1 Hz optical pulsing. Source data are provided as a Source Data file.

7. Growth methods and surface characteristics of Element Six's diamond substrates

The polycrystalline diamond substrates used in our experiments were sourced from Element Six. According to the company's *Diamond Handbook 2024*, these substrates were grown via microwave plasma-enhanced chemical vapor deposition (MPCVD). The growth conditions are created by thermal dissociation of hydrogen, and a gaseous source of carbon in plasma, with a gas temperature above 2000°C.

We utilized polycrystalline diamond substrates from *Element Six*'s TM180 series. According to the manufacturer's specifications, these substrates demonstrate: Thermal conductivity >1800 W/m·K at 300 K, Thermal conductivity >1500 W/m·K at 425 K, Polished surface roughness (Ra) <20 nm.

Supplementary Table 1 | TM180 Polycrystalline CVD Diamond specification table (*Diamond Handbook 2024*)

**Property**	**Value**	**Unit**
**Thermal conductivity**		
@300 K	>1800	$W \cdot m^{-1} \cdot K^{-1}$
@425 K	>1500	$W \cdot m^{-1} \cdot K^{-1}$
**Specific heat capacity**	520	$J \cdot kg^{-1} \cdot K^{-1}$
**Density**	3.52	$\times 10^3$ $kg \cdot m^{-3}$
**Volumetric heat capacity**	$\sim 1.83 \times 10^6$	$J \cdot m^{-3} \cdot K^{-1}$
**Thermal expansion coefficient**		
@300 K	1.0 ± 0.1	$ppm \cdot K^{-1}$
@1000 K	4.0 ± 0.1	$ppm \cdot K^{-1}$
**Thermal diffusivity**	> 10.0	$cm^2 \cdot s^{-1}$
**Fracture toughness**	5.3 – 7.0	$MPa \cdot m^{0.5}$
**Young's modulus**	1050	GPa
**Poisson's ratio**	0.1	—
**Resistivity (bulk)**	10^{12}	Ω·cm
